# Analysis of the functional repertoire of a mutant form of survivin, K129E, which has been linked to lung cancer

**DOI:** 10.1186/s12935-014-0078-8

**Published:** 2014-08-19

**Authors:** Aysha M Aljaberi, Jamie RM Webster, Sally P Wheatley

**Affiliations:** 1 School of Life Sciences, University of Nottingham, Queen’s Medical Centre, Nottingham NG7 2UH, UK; 2 Present address: Protein Expression Facility, School of Cancer Sciences, University of Birmingham, Vincent Drive, Birmingham B15 2TT, UK

**Keywords:** Survivin, Borealin, Mitosis, Cytokinesis, Apoptosis, Cancer, Polymorphism

## Abstract

**Background:**

Survivin is a protein that is normally present only in G2 and M-phases in somatic cells, however, in cancer cells, it is expressed throughout the cell cycle. A prosurvival factor, survivin is both an inhibitor of apoptosis and an essential mitotic protein, thus it has attracted much attention as a target for new oncotherapies. Despite its prevalence in cancer, reports of survivin mutations have mostly been restricted to loci within its promoter, which increase the abundance of the protein. To date the only published mutation within the coding sequence is an adenine > guanine substitution in exon 4. This polymorphism, which was found in a cohort of Korean lung cancer patients, causes a lysine > glutamic acid mutation (K129E) in the protein. However, whether it plays a causative role in cancer has not been addressed.

**Methods:**

Using site directed mutagenesis we recapitulate K129E expression in cultured human cells and assess its anti-apoptotic and mitotic activities.

**Results:**

K129E retains its anti-apoptotic activity, but causes errors in mitosis and cytokinesis, which may be linked to its reduced affinity for borealin.

**Conclusion:**

K129E expression can induce genomic instability by introducing mitotic aberrations, thus it may play a causative role in cancer.

## Background

**Figure 1:**
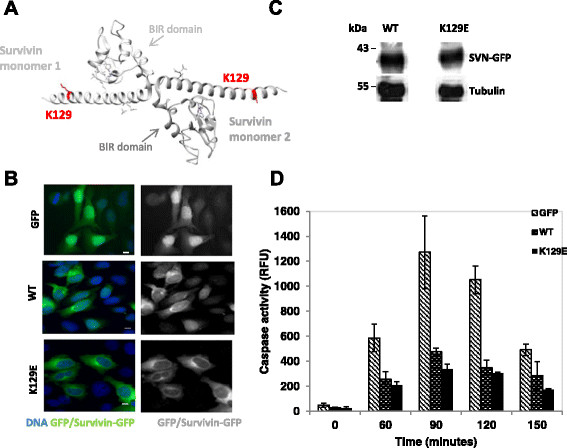
**K129E is cytoplasmic and can inhibit apoptosis. (A)** 3D ribbon model of the survivin homodimer constructed from structure 1F3H using the UCSF Chimera package (http://www.cgl.ucsf.edu/chimera). The survivin monomers are shown in light and mid-grey. Residue K129 and its side chain is highlighted in red. **(B)** HeLa cells in interphase stably expressing GFP, WT survivin, or K129E as indicated in green, counterstained with DAPI to highlight the nucleus (blue), bar 5 μm. Both WT and K129E localise predominantly in the cytoplasm. **(C)** An immunoblot showing WT and K129E are expressed at similar levels in whole cell extracts prepared from asynchronous populations. Tubulin indicates equality in loading. **(D)** HeLa cells expressing GFP (control), WT, or K129E were treated with TRAIL for 0–150 minutes then a caspase-3 activity performed on whole cell extracts to indicate apoptosis. Caspase activity was measured spectrophotometrically and is plotted in relative fluorescent units (RFU).

Survivin is a cancer relevant protein that is involved in cell division and cell death. Described as the “fourth most upregulated transcript” in all cancers [1], its increased abundance is linked at the clinical level to tumour resistance to chemo and radiation treatments, and its presence predicts a poor prognosis for the patient [2],[3]. Survivin expression is regulated by multiple transcription factors. Briefly, within its promoter there are multiple Sp1 sites, three cell cycle dependent elements (CDE), and a cell cycle homology region (CHR) site, reviewed in [4]. It is known to be repressed by p53 [5], and upregulated by NF-kB and GATA-1, and several others are implicated in promoting its expression including the E2F family and STAT3 [4]. Despite its prevalence in cancer, to date the only single nucleotide polymorphisms SNP reported within the human survivin cDNA, in the literature, is an adenine to guanine substitution in exon 4. Upon translation this point substitution causes an amino acid change in the C-terminal alpha helix of the protein such that a glutamic acid is selected as residue 129 instead of a lysine, hereinafter denoted as K129E (see Figure 1A). This SNP was one of eight found in a population of lung cancer patients from Korea [6], the seven others reported in this study occurred within the CDE/CHR homology domain in the promoter, or the 3′UTR, see also [7],[8]. At only 420 base pairs in size, it is perhaps not surprising that SNPs within the coding region of the survivin gene are rare, but as this tiny protein is a prosurvival factor with multiple binding partners and multiple roles, a charge substitution in a conserved domain is likely to impact on its function. Nevertheless, whether K129E is an inconsequential mutation or one that could contribute to the diseased state has not been addressed at the molecular level. Here we recapitulate expression of this mutation in cultured cells to assess whether it is likely to play a causative role in cancer.

## Results and discussion

### K129E is cytoplasmic and can inhibit apoptosis

To begin this investigation, site directed mutagenesis was used to create a point mutation in the human survivin gene, which translates to a K129E substitution in the survivin protein. This version was C-terminally tagged with green fluorescent protein (GFP) and cloned into the mammalian expression vector, pcDNA3.1. HeLa cell lines stably expressing GFP (control), wild type GFP tagged survivin (WT) or K129E, were established by transfection followed by G418 selection, and FACS sorting, and used in all subsequent experiments. As shown in Figure 1B, while GFP alone was found throughout interphase cells, both WT and K129E were predominantly cytoplasmic. We also noted by immunoblotting that the level of expression of the ectopic survivin-GFP variants in these stable lines was comparable (Figure 1C).

To determine whether K129E compromises the anti-apoptotic activity of survivin, exponentially growing HeLa cells expressing GFP, WT or K129E were treated with TRAIL for 0, 30, 60 or 90 minutes and whole cell extracts prepared from these populations were assessed for caspase-3 activity using a DEVD-cleavage assay. As shown in Figure 1D, K129E reduced the level of caspase-3 activity as effectively as WT overexpression indicating that this mutant retains the ability to inhibit apoptosis.

As a member of the inhibitor of apoptosis (IAP) family of proteins, survivin has a baculovirus inhibitor of apoptosis repeat (BIR) domain comprising residues ~ 15–80 (see Figure 1A), a domain associated with caspase inhibition [9]. Exclusion from the nucleus is also an important factor in the activity of survivin as an anti-death agent [10]–[12]. Thus as K129 is positioned out with the BIR domain, in the C-terminus, and K129E is predominantly cytoplasmic, it is not surprising that K129E retains that ability to inhibit apoptosis.

### K129E causes a delay in mitosis

**Figure 2:**
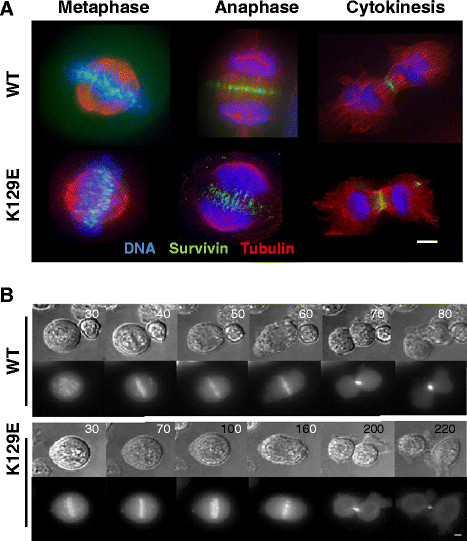
**K129E in mitosis. (A)** Fluorescence images of cells in metaphase, anaphase and cytokinesis, with WT or K129E shown in green, microtubules immunostained (red) and chromosomes counterstained with DAPI (blue). K129E localises similarly to WT at all stages. **(B)** Still images taken from time lapse experiment recording mitosis in cells expressing WT or K129E. Cells were treated for 3 h with DMA to induce monopolar spindles with chromosomes attached in a syntelic configuration, and time (minutes) since DMA removal is indicated in top right corner. Upper panels DIC, lower panels GFP. Cells expressing K129E divide more slowly than those expressing WT. Scale bars 5 μm.

Having established that K129E does not interfere with the IAP activity of survivin, we next asked whether it affected its role in mitosis. To address this we first examined its localisation in fixed cells expressing GFP tagged WT or K129E using immunofluorescence with anti-tubulin antibodies to reveal the microtubules, and DAPI to highlight the chromosomes. Just like the WT form, K129E localized to the centromeres in prometaphase, the midzone microtubules in anaphase, and the midbody in cytokinesis (Figure 2A).

During early mitosis and as part of the chromosomal passenger complex (CPC), survivin is required to facilitate correction of chromosomes that have attached to the mitotic spindle in an erroneous fashion [13],[14]. To test whether K129E compromises this essential mitotic role an “error correction” assay was performed. Briefly, cells were arrested in mitosis with monopolar spindles and syntelically orientated chromosomes using the Eg5 inhibitor dimethylenastron (DMA). Upon release from DMA the subsequent mitotic events were monitored by live imaging (Figure 2B). In this assay approximately 80% of cells expressing K129E (N = 52) were able to correct erroneously attached chromosomes, which was similar in outcome to those expressing WT (N = 20). However on average they took 60 minutes longer to achieve alignment than their WT counterparts (WT: 200 ± 50 minutes, K129E: 260 ± 35 minutes). These data suggest that K129E can correct maloriented chromosomes, but is less efficient at doing so than WT. They also suggest that the spindle assembly checkpoint is operational, and consistent with this, BubR1 is recruited to the centromeres of DMA arrested K129E expressing cells (data not shown).

### When expressed alone K129E cannot support cell proliferation

**Figure 3:**
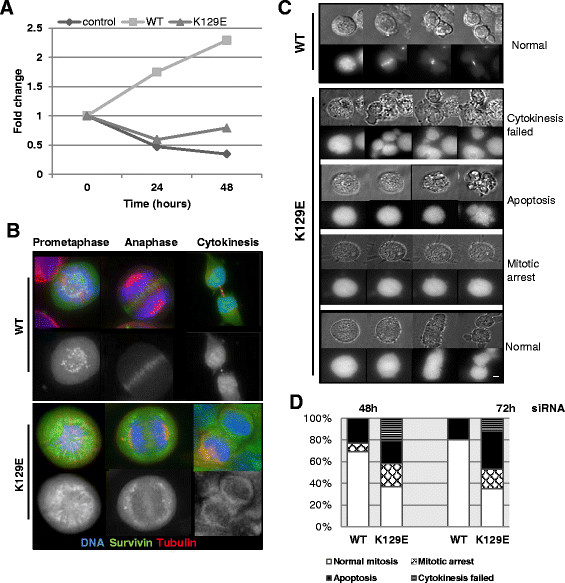
**K129E expressed alone cannot support mitosis. (A)** Cells were treated with control or survivin specific siRNA as described in Wheatley et al. [22], and cell number monitored by trypan blue exclusion. Fold difference between control treatment and survivin siRNA-specific treatment is plotted, and is representative of three independent experiments. **(B)** Immunofluorescence images of formaldeyde fixed cells in prometaphase, anaphase and cytokinesis 72 h after depletion of the endogenous protein showing WT or K129E (green), microtubules (red) and chromosomes (blue). Scale bar 5 μm. **(C)** Cells depleted of endogenous survivin were then arrested in mitosis by 3 h treatment with DMA and imaged in DIC (upper panels) and GFP (lower panels) to determine their fate post-release from mitotic arrest, as in Figure 2B. **(D)** Quantitation of cell fates post-release from DMA and depleted of endogenous survivin for 48 h or 72 h.

In all experiments described above, the wild type protein was present. To address the consequences of expressing only K129E during cell division, we eliminated the endogenous protein by siRNA and repeated our assays. Depletion of the endogenous protein and resistance of the ectopic WT and K129E forms was verified by immunoblotting 48 h post-transfection (data not shown) and the consequences of this action to cell proliferation monitored using a trypan blue assay. As shown in Figure 3A control cells expressing only GFP stopped growing 24 h post-siRNA, while cells expressing the siRNA resistant WT survivin continued to thrive. K129E, however, was unable to restore proliferation. Moreover, as shown in Figure 3B, under these conditions, K129E was no longer able to localise to the centromeres in prometaphase, or the central spindle in anaphase. It was also absent from the midbody and binucleated cells were commonly seen. Next, we repeated the live imaging error-correction assay in the siRNA treated cells. Upon release from this imposed monopolar-syntelic chromosome configuration cells expressing K129E had one of four outcomes (Figure 3C): 1. cytokinesis failure, in which case they divided abnormally or became binucleated; 2. apoptosis; 3. mitotic arrest for the 12 h duration of the experiment; or 4. normal mitosis. Quantitation of the outcome cell populations monitored for 12 h post-DMA release that had been depleted of endogenous survivin for 48 h or 72 h are presented in Figure 3D.

### K129E has reduced affinity for borealin

**Figure 4:**
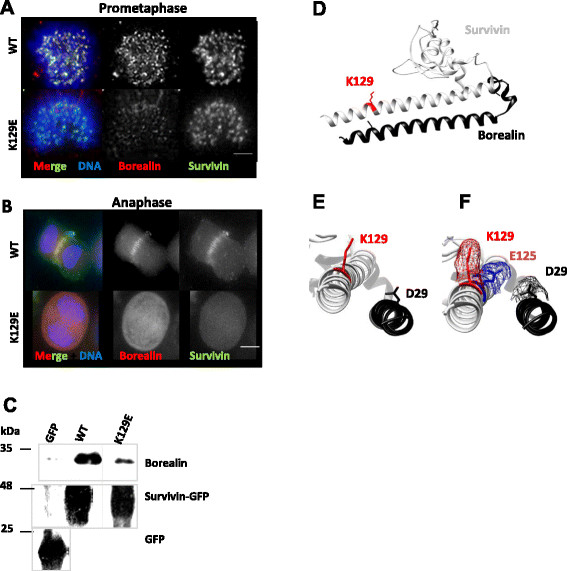
**K129E reduces the affinity of survivin for borealin. (A)** HeLa cells expressing WT or K129E (green), depleted of endogenous survivin were fixed with formaldehyde and probed to localise borealin (red) at the centromeres of prometaphase cells or **(B)** at the anaphase spindle. **(C)** Immunoblot of borealin co-immunoprecipitated with anti-GFP antibodies from lysates prepared from cells ectopically expressing GFP, WT or K129E. **(D-F)** 3D ribbon models of survivin (grey) bound to the NH_2_ terminus of borealin (black) constructed from structure 2RAW using UCSF Chimera [20]. K129 and its side chain are highlighted in red. **(E, F)** Magnified and rotated models of the C-terminus of survivin bound to borealin from **(D)**, with the side chain of D29 of borealin (black), and **(F)** an upstream glutamic acid (E125^svn^) indicated in blue mesh to demonstrate how this charge change could affect the proximity of K129^svn^ (red mesh) and D29^bor^ (black mesh).

Although not engaged in homodimerisation, the C-terminal amphipathic helix of survivin is central to the integrity of the essential mitotic CPC. The CPC is composed of four proteins, aurora-B kinase, which is the enzymatic component, and three other proteins, INCENP, borealin and survivin. The CPC components are mutually dependent upon each other for correct localisation and function during mitosis, reviewed in [15],[16]. The latter three proteins interact via long alpha-helices to form an interdependent triple helical bundle [17]. Disrupting the integrity of this core helical bundle has been shown to prevent the CPC from transferring from the centromeres to the central spindle in anaphase [17]. Thus our next question was whether K129E affected the helical interaction between survivin and borealin. To answer this we first immunoprobed cells depleted of endogenous survivin, expressing only K129E, with antibodies to borealin. As shown in Figure 4A, discrete borealin and WT survivin foci were present in prometaphase cells in the WT population, however, in accordance with the more diffuse localisation of K129E, and its reduced presence at centromeres, borealin staining was also diminished at the centromeres of K129E cells. Moreover in anaphase, borealin did not transfer to the central spindle (Figure 4B). These data are consistent with reports from other groups that have demonstrated the importance of the C-terminus of survivin in transfer to the central spindle and the completion of cytokinesis [18],[19]. Finally, we performed an immunoprecipitation assay with anti-GFP antibodies from cell lines expressing GFP, WT or K129E, and interrogated the precipitates for the presence of borealin. As shown in Figure 4C, abundant borealin co-immunoprecipitated with WT survivin, but this was greatly reduced in the K129E lysate. Collectively these data suggest that the mitotic defects manifested in cells expressing K129E are likely due to its reduced affinity for borealin.

From the crystal structure K129 lies in close proximity to D29 of borealin, where it is well positioned to form an attractive electrostatic association (Figure 4D and E). A reverse charge substitution to glutamic acid (K129E) would prevent this attraction, or even cause a repulsive force to the NH_2_ terminal helix of borealin, which could disrupt the helical association. In Figure 4F a glutamic acid (E125) four residues upstream of K129, has been highlighted in blue to indicate how the angle of the glutamic acid side chain differs from that of lysine placed similarly in the helix. This change in proximity of the side chains, together with the charge reversal could conceivably drive the two helices apart. Interestingly K129 can be acetylated, a post-translational modification that has been shown to affect its interphase localisation [21]. The substitution K129E would abrogate acetyl-regulation, however, whether acetylation of this site is mitotically relevant has not yet been addressed.

## Conclusions

In conclusion, the aim of this study was to determine whether the SNP that causes mutation of the survivin protein such that K129 is substituted for glutamic acid, could play a causative role in cancer. In the original paper by Jang et al. [6], the risk of having lung cancer appeared greatest in individuals who had a combination of SNPs with one allele causing K129E substitution, and the second allele having a point mutation in the promoter region of the genomic DNA. Although their genetic analysis suggested that the more influential SNP was within the promoter, the data herein presented suggest that this reverse charge substitution in the protein may promote the diseased state by decreasing the affinity of survivin for borealin, thus causing mitotic defects and increasing genomic instability.

## Methods

Unless otherwise indicated, tissue culture reagents were obtained from Invitrogen, and all other reagents from Sigma-Aldrich.

### Molecular cloning

The point mutation, K129E, was generated by site-directed mutagenesis using the 5′ primer: GAGGAAACTGCGGAGAAAGTGCGCCGTG; and the 3′ complementary primer: CACGGCGCACTTTCTCCGCAGTTTCCTC (Eurofins, MWG Operon), VENT polymerase (NEB), dNTPs and wild-type human survivin cDNA bearing a siRNA resistant mutation at C54G in pBluescript as a template [22], according to instructions in the Stratagene Quickchange II kit (Agilent Technologies). The parental cDNA was digested with DpnI and nascent cDNA transformed into competent DH5 alpha *E. coli* cells. Constructs were then subcloned with EcoRI and HindIII restriction enzymes (NEB) into pcDNA3.1 (Invitrogen), with a COOH-terminal GFP tag for expression in mammalian cells, and the sequence verified prior to use.

### Cell culture

HeLa cells were cultured at 37°C and 5% CO_2_ humidified incubator in Dulbecco’s Modified Eagle’s Medium (DMEM) supplemented with 10% HyClone bovine serum (FBS), L-glutamine (2 mM), 1% penicillin-streptomycin and 1% fungizone. To create cell lines stably expressing the desired constructs, cells were transfected with pcDNA3.1 constructs using FuGENE 6 (Roche Diagnostics) diluted in Opti-MEM, and then cultured in antibiotic free medium. 24 h post-transfection, G418 (50 μg/ml) was added to select for positive transformants, and GFP positive cells were harvested 7–10 days later by FACS sorting (MoFLo).

### Immunoblotting

Standard procedures were used for SDS-PAGE (12%) and immunoblotting with 0.22 μm nitrocellulose (PALL). To reveal both exogenous and endogenous survivin-GFP, nitrocellulose membranes were probed with anti-survivin antibodies (1/1000, in house), anti-tubulin (B512, 1/2000, Sigma) as a loading control, and anti-borealin (1/1000, in house) all diluted in PBS with 5% milk and 0.1% Tween 20. Horse-radish peroxidise-conjugated secondary antibodies (DAKO, 1/2000), enhanced chemiluminescence (ECL; GeneFlow) and X-ray film (GE Healthcare) were used to detect bands using standard procedures.

### Apoptosis assay

To assess apoptosis 10^5^ cells were seeded into 24-well plates, then incubated with 250 ng/ml TRAIL for 0, 60, 90, 120, or 150 minutes. Cells were lysed in 150 μl of mammalian protein extraction reagent (MPER, Pierce) and 1 mM EDTA. Proteases were inhibited with pepstatin A and 4-(2-aminoethyl)-bensesulfonyl fluoride (AEBSF) both at 1 μg/ml. 40 μl of each cell lysate was incubated in a 96-well plate with 200 μl of caspase assay buffer (20 mM HEPES at pH7.5, 10% glycerol, 1 mM dithiothreitol) and caspase-3 fluorogenic substrate Ac-DEVD-AMC (BioMol Research Labs) at 37°C for 1 hour. Cleavage of this tetrapeptide substrate was measured spectrophotometrically using a FLUOstar Galaxy Spectrophotometer (BMG Lab technologies) set at 390 nm excitation, and 450 nm emission.

### Immunofluorescence microscopy

Cells were cultured on polylysine coated coverslips, fixed with 4% formaldehyde, and permeablized using 0.15% Triton-x-100 in PBS. They were then blocked with 1% BSA in PBS and immunoprobed with primary antibodies for 1 h at room temperature, then incubated with texas-red (1/200, Vector) or Cy5-conjugated secondary antibodies (1/1000, AbCam). Mitotic spindles were revealed with tubulin (B512, 1/2000, Sigma), borealin with a polyclonal rabbit antibody (1/500, in house), and BubR1 with a polyclonal sheep anti- BubR1 antibody (1/1000, gift from Prof. Stephen Taylor, Manchester). Samples were counterstained with DAPI to reveal DNA, and mounted with Vectashield (Vector Laboratories). Images of fixed cells were acquired using an inverted (Olympus IX71, Delta Vision Elite) microscope fitted with 20x (NA 0.85, oil) or 60x (NA1.4, oil) objectives using DeltaVision software (G.E.Healthcare) and a Coolsnap HQ^2^ camera (Photometrics). For high magnification images two-dimensional projections were created from deconvolved Z-stacks (0.3 μm sections) and then prepared using Adobe Photoshop.

### Live imaging

Exponentially growing cells were treated with 2 μM dimethylenastron (DMA, Enzo Life Sciences) for 3 h then harvested onto 35 mm, poly-l-lysine coated, glass-bottomed live image dishes (Willco) by mitotic shake-off. DMA was then washed out and cells monitored using Delta Vision Elite microscope fitted with 40 × (NA 0.95, air) objective on cells grown in 35 mm glass-bottomed dishes (Willco) in phenol-red free CO_2_ independent medium. Z-sweeps of 0.5 μm sections were acquired every 3 or 5 minutes of both GFP and differential interference contrast channels. Subsequent analysis was carried out using Volocity software (Improvision) and still images prepared with Adobe Photoshop.

### RNAi

HeLa cells stably expressing GFP or GFP tagged survivin constructs were seeded at 5 × 10^4^ cells per well of a 24-well plate immediately before transfection with 60 pmol of control or survivin specific siRNA using HiPerFect (Qiagen) in antibiotic free medium. Note that WT and K129E constructs were resistant to RNAi targeting as they contain the silent mutation, G54C [22]. Cell proliferation was assessed using the trypan blue exclusion method and a haemocytometer.

### Immunoprecipitation

3 × 10^6^ cells were harvested in RIPA buffer (20 mM Tris–HCl at pH8, 137 mM NaCl, 0.5 mM EDTA, 10% Glycerol, 1% NP40, 0.1% SDS, 0.1% deoxycholate, 2 mM β-glycerophosphate) with 2U benzonase nuclease, supplemented with 2 mM MgCl_2_ and incubated for 30 minutes at 4°C. The resulting whole cell lysates were then precleared with protein A/G beads (Pierce) to reduce non-specific protein binding. After preclearing, each lysate was incubated with 2 μg of anti-GFP antibody (Roche) per 1 mg of sample and incubated overnight at 4°C with rotation. Next, 20 μl of 50% A/G bead slurry was added and incubated at 4°C for 2 h. Beads with associated immunoprecipitated proteins were then washed for 15 minutes at 4°C sequentially in RB1: 50 mM Tris–HCl, 0.15 M NaCl, 0.1% EDTA, 0.1% NP40 pH 8; RB2: 50 mM Tris–HCl, 0.15 M NaCl, 0.1% NP40 pH 8; and RB3: 50 mM Tris–HCl, 0.1% NP40, pH 8. After washing beads were boiled for 2 minutes in SDS sample buffer to isolate immunocomplexes and analysed by immunoblotting.

## Abbreviations

BIR: Baculovirus inhibitor of apoptosis repeat

CPC: Chromosomal passenger complex

DIC: Differential interference contrast

DMA: Dimethylenastron

FACS: Fluorescent activated cell sorting

GFP: Green fluorescent protein

IAP: Inhibitor of apoptosis protein

SNP: Single nucleotide polymorphism

WT: Wild type

## Competing interests

The authors declare that they have no competing interests.

## Authors’ contributions

The project was conceived and directed by SPW. JRMW carried out the DNA manipulations and provided technical support for the project. All other experiments were performed by AA. SPW and AA wrote the paper. All authors read and approved the final manuscript.
